# Association between dietary carotenoid intake and vertebral fracture in people aged 50 years and older: a study based on the National Health and Nutrition Examination Survey

**DOI:** 10.1007/s11657-025-01508-5

**Published:** 2025-03-15

**Authors:** Yuchen Zheng, Wenyu Zhou, Jian Zhang, Tao Lan, Rui Zhang

**Affiliations:** Department of Spine Surgery, Futian District, Shenzhen Second People’s Hospital, The First Affiliated Hospital of Shenzhen University, No. 3002, Sungang West Road, Shenzhen, 518035 Guangdong China

**Keywords:** Dietary carotenoids, β-Carotene, Vertebral fracture, Gender, The National Health and Nutrition Examination Survey

## Abstract

**Summary:**

This study discussed the association between dietary intake of carotenoids and vertebral fractures by analyzing the target data, concluding a result of negative association and providing valuable information on vertebral fracture.

**Objective:**

This study is to explore the association between dietary intake of carotenoids and vertebral fractures.

**Methods:**

Data of individuals aged ≥ 50 years from the 2013–2014 National Health and Nutrition Examination Survey (NHANES) database were used in this cross-sectional study. Information on dietary carotenoid intake was obtained from the first 24-h dietary recall interview. Vertebral fractures were assessed using dual-energy x-ray absorptiometry (DXA). The weighted multivariable logistic regression model was established to assess the association between dietary carotenoid intake and risk of vertebral fracture. Subgroup analysis of fracture history and menopausal status was performed for further analysis of this relationship.

**Results:**

Of the 2053 eligible study subjects, there were 1021 men and 1032 women. Increased β-carotene intake was associated with decreased odds of vertebral fracture in women (odds ratio (OR) = 0.77, 95% confidence interval (CI) 0.60–0.99, *P* = 0.047), after adjusting for covariates. In men without a history of fracture, lutein and zeaxanthin intake was negatively associated with increased odds of vertebral fracture (OR = 0.70, 95% CI 0.50–0.99, *P* = 0.048). In women without a history of fracture, increased β-carotene intake was associated with decreased odds of vertebral fracture (OR = 0.78, 95% CI 0.61–0.99, *P* = 0.047). In postmenopausal women, β-carotene intake was also negatively associated with increased odds of vertebral fracture (OR = 0.77, 95% CI 0.60–0.99, *P* = 0.048).

**Conclusion:**

This research concludes a negative association between dietary carotenoid intake, especially β-carotene, and vertebral fractures in women, revealing a potential dietary prevention tactic for vertebral fractures in the future.

**Supplementary Information:**

The online version contains supplementary material available at 10.1007/s11657-025-01508-5.

## Introduction

Vertebral fractures, a common type of fracture in people over the age of 50, are related to skeletal fragility and osteoporosis, which can cause acute and chronic back pain, affecting patients’ quality of life and survival [1, 2]. Although the definition of vertebral fracture may vary between studies, it is consistently reported that vertebral fractures affect at least 20% of people over the age of 50 [3]. In postmenopausal women, the incidence of vertebral fractures can increase by more than 50% with age [4]. Therefore, it is of great significance to identify factors associated with vertebral fractures and to conduct early interventions to lower the risk of vertebral fractures and subsequently improve patients’ quality of life.

Oxidative stress is an important pathogenic factor contributing to bone loss [5]. The intake of dietary antioxidants may be of important clinical value in improving bone loss and reducing the risk of fragility fractures [6]. Carotenoids are a group of fat-soluble natural pigments acting as antioxidants in the body. Over 95% of the total carotenoids in human blood are α-carotene, β-carotene, β-cryptoxanthin, lycopene, and lutein and zeaxanthin [7]. Evidences have shown that carotenoids can stimulate bone formation and inhibit bone resorption [8, 9]. Studies in different populations illustrated that the high intake of specific carotenoids is positively associated with bone health and a reduced risk of osteoporosis [10, 11]. A previous long-term cohort study found a protective effect of higher carotenoid intake on hip fracture [10], but the relationship between carotenoids and vertebral fractures is still unclear.

Herein, the objective of this study is to explore the association between dietary carotenoid intake and vertebral fractures in people aged 50 years and older in America. The hypothesis is that dietary carotenoid intake is negatively associated with the odds of vertebral fracture in this population. Subgroup analyses of sex and fracture history were also performed to evaluate whether the association between dietary carotenoids and vertebral fractures varied in different subpopulations.

## Materials and methods

### Study setting and population

This cross-sectional study extracted data from the 2013–2014 National Health and Nutrition Examination Survey (NHANES) database. NHANES is a series of multi-stage surveys performed by the Centers for Disease Control and Prevention (CDC) to assess the health and nutritional status of the nationally representative population in America. The survey combines interviews and physical examinations, and more detailed information on this survey has been presented elsewhere: https://www.cdc.gov/nchs/nhanes/about_nhanes.htm.

This study included individuals aged ≥ 50 years. Participants (1) without the assessment of vertebral fracture, (2) without complete data on dietary carotenoid intake, (3) complicated by malignant tumors, (4) using anti-osteoporosis drugs, or (5) men consuming less than 500 kcal or more than 8000 kcal, while women consuming less than 500 kcal or more than 5000 kcal, were excluded. Since the data used are publicly available and de-identified, ethical approval by the institutional review board was exempt.

### Assessment of dietary carotenoid intake

In the NHANES, information on dietary intake of carotenoids was obtained via two 24-h dietary recall interviews. The first dietary recall interview was conducted in person in the Mobile Examination Center (MEC). A set of measuring guides (various glasses, bowls, mugs, etc.) was available in the MEC dietary interview room for the participants to use to report food quantities. The second dietary recall interview was conducted by telephone or mail 3–10 days after the first interview.

Five different dietary carotenoids were evaluated in this study, including α-carotene, β-carotene, β-cryptoxanthin, lycopene, and lutein and zeaxanthin. The dietary data we used were the mean of two 24-h reviews, which included dietary intake and dietary supplements. Dietary carotenoids were further adjusted by energy intake: energy-adjusted carotenoids (µg/1000 kcal) = dietary carotenoid intake/energy intake × 1000, indicating the micrograms of carotenoids per 1000 kcal of energy. Additionally, according to the conversion method of retinol activity equivalent (RAE), the energy-adjusted carotenoid vitamin A was calculated as follows: energy-adjusted carotenoid vitamin A = 1/12 × β-carotene + 1/24 × α-carotene + 1/24 × β-cryptoxanthin; and the total carotenoid = α-carotene + β-carotene + β-cryptoxanthin + lycopene + lutein and zeaxanthin.

### Vertebral fracture diagnosis

At MEC, dual-energy x-ray absorptiometry (DXA) was used to diagnose vertebral fractures by conducting a lateral scan of the thoraco-lumbar spine. All scans were analyzed by Optasia Spinalizer software using Genant’s semiquantitative (SQ) technique. Grade 0 was represented as normal status, Grade 1 as mild deformity, Grade 2 as moderate deformity, and Grade 3 as severe deformity. Grade 1 and above were diagnosed as vertebral fracture [12]. Besides, considering the influence from mild deformity, we categorized people with non-vertebral fractures or Grade 1 into one group, and those with Grade 2 or 3 into another group for sensitivity analysis.

### Covariates

The following data were extracted from the database as potential covariates: age (years), gender, race, poverty income ratio (PIR), education, smoking, alcohol consumption, a history of fracture, body mass index (BMI, kg/cm^3^), waist circumference (cm), femoral neck bone mineral density (BMD) (g/cm^2^), Mediterranean score, physical activity (MET·min), Healthy Eating Index (HEI)−2010, diabetes, parental fracture, glucocorticoid use, calcium (mg), and total energy intake (kcal).

Race was classified as Mexican American, non-Hispanic Black, non-Hispanic White, other Hispanic race, and other race [13]. Education was divided into college graduate, high school/General Education Development (GED), less than high school, and some college [14]. A history of fracture was defined as broken or fractured a hip. Participants with fasting blood glucose ≥ 7.0 mmol/L or hemoglobin A1c (HbAlc) ≥ 6.5% or self-reported diabetes or receiving hypoglycemic treatment were regarded to have diabetes [15]. For BMI as a categorical variable, < 18.5 kg/m^2^ was considered underweight, 18.5–24.9 kg/m^2^ as normal weight, 25.0–29.9 kg/m^2^ as overweight, and ≥ 30.0 kg/m^2^ as obese [16]. Women were categorized as menopausal if they answered “Menopause” to the question “What is the reason that you have not had a period in the last 12 months?”.

### Statistical analysis

The Kolmogorov–Smirnov test was used to examine the normality of quantitative data. Measurement data with normal distribution was described by mean (standard error) (mean (SE)), and the independent sample *t*-test was utilized to compare the characteristics between the two groups. Counting data were expressed by the number of cases and constituent ratio (*n* (%)), and the chi-square test was used for inter-group comparison. In NHANES, special sample weights are created to account for the complex survey design (including oversampling), survey non-response, and post-stratification adjustment to match total population counts from the Census Bureau. Since the dietary information used in this study was the average values of two 24-h dietary recalls, the “dietary day two sample weight (WTDR2D)” was used for sample weights, as recommended by NHANES.

Variables with statistical significance in the weighted univariable logistic regression model and variables with clinical significance were selected as adjustment variables. Correlations among dietary carotenoids were also assessed and considered in multivariate model adjustment. The weighted multivariable logistic regression model was established to assess the association between dietary carotenoid intake and the risk of vertebral fracture. Variables with missing values in this study were imputed with the use of multiple imputation. Multiple imputation, a method of handling missing values based on repeated simulation, is mainly completed by the “mice” package in R software (Supplementary Table 1). Considering that the application of imputation may affect the association between dietary carotenoids and vertebral fracture, sensitivity analysis was carried out by comparing the data before and after imputation (Supplementary Table 2). Subgroup analysis was further performed in terms of menopausal status and fracture history to analyze the association between dietary carotenoids and vertebral fracture in different subgroups. Odds ratios (ORs) with 95% confidence intervals (CIs) were calculated to evaluate the effect size, and *P* < 0.05 indicated statistical significance.

Data cleaning (including missing value statistics) and missing value interpolation were completed using Python 3.7.4 (Python Software Foundation, DE, USA). Sensitivity analysis, difference comparison, statistical modeling, and subgroup analysis were conducted by SAS 9.4 (SAS Institute Inc., Cary, NC, USA), and result visualization was subject to R 4.2.1 (R Foundation for Statistical Computing, Vienna, Austria).

## Results

### Participant characteristics

**Fig. 1:**
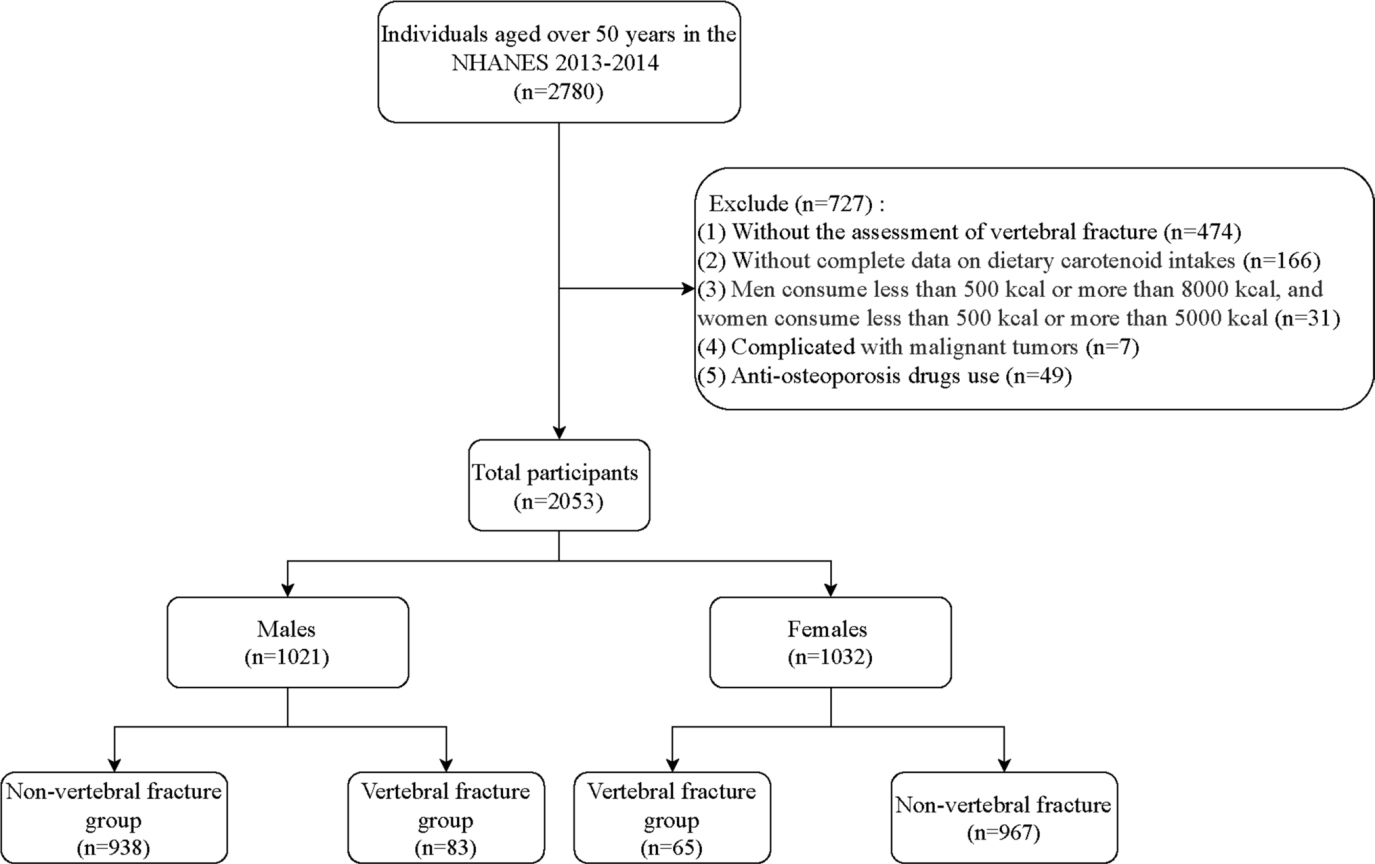
Flow chart of participant selection. Notes: NHANES, the National Health and Nutrition Examination Survey

Initially, 2780 individuals aged 50 years and older were included in this study. After excluding people without the assessment of vertebral fracture (*n* = 474), incomplete data on dietary carotenoid intake (*n* = 166), and complicated with malignant tumors (*n* = 7), and man intake ≤ 500 kcal or > 8000 kcal and feman ≤ 500 kcal or > 5000 kcal (*n* = 31), and use of anti-osteoporosis drugs (*n* = 49), 2053 were eventually involved, including 1021 men and 1032 women. The flow chart of participant selection is illustrated in Fig. 1. In both genders, the average age of patients in the vertebral fracture group was significantly higher than that in the non-vertebral fracture group (all *P* < 0.05). In men participants, PIR, BMD of femoral neck, and physical activity in the non-vertebral fracture group were significantly higher than those in the vertebral fracture group (all *P* < 0.05). In women, femoral neck bone mineral density, waist circumference, β-carotene, vitamin A, and total carotenoids in the non-vertebral fracture group were higher than those in the vertebral fracture group (all *P* < 0.05). Table 1 exhibits the characteristics of these participants.

**Table 1:** Characteristics of the included subjects

Variables	Male (*n* = 1021)				Female (*n* = 1032))			
	Non-vertebral fracture group (*n* = 938)	Vertebral fracture group (*n* = 83)	Statistics	*P*	Non-vertebral fracture group (*n* = 967)	Vertebral fracture group (*n* = 65)	Statistics	*P*
Age, years, mean (SE)	61.56 (0.43)	67.66 (1.75)	*t* = − 3.38	0.004	62.24 (0.36)	68.04 (1.87)	*t* = − 3.46	0.004
Race, *n* (%)			*χ*^2^ = 8.961	0.062			*χ*^2^ = 3.891	0.421
Mexican American	129 (5.93)	10 (9.90)			105 (4.57)	4 (1.83)		
Non-Hispanic Black	222 (10.86)	8 (4.42)			188 (10.45)	10 (5.80)		
Non-Hispanic White	406 (74.86)	57 (71.71)			473 (73.89)	42 (83.22)		
Other Hispanic	78 (3.38)	2 (1.28)			100 (4.05)	3 (2.50)		
Other race	103 (4.97)	6 (12.69)			101 (7.04)	6 (6.66)		
PIR, mean (SE)	3.41 (0.14)	2.70 (0.25)	*t* = 2.88	0.011	3.18 (0.11)	2.86 (0.32)	*t* = 0.98	0.343
Education, *n* (%)			*χ*^2^ = 0.769	0.857			*χ*^2^ = 3.992	0.262
College graduate	258 (38.85)	19 (35.11)			216 (29.03)	18 (39.39)		
High school/GED	205 (18.55)	21 (21.60)			227 (21.18)	20 (21.70)		
Less than high school	244 (16.76)	16 (13.18)			197 (11.75)	14 (15.78)		
Some college	231 (25.84)	27 (30.12)			327 (38.04)	13 (23.13)		
Smoking, *n* (%)			*χ*^2^ = 3.055	0.080			*χ*^2^ = 0.067	0.796
No	402 (48.12)	26 (31.21)			575 (57.07)	39 (60.24)		
Yes	536 (51.88)	57 (68.79)			392 (42.93)	26 (39.76)		
Alcohol consumption, *n* (%)			*χ*^2^ = 0.314	0.576			*χ*^2^ = 0.646	0.422
No	142 (10.94)	11 (8.59)			427 (34.50)	28 (41.71)		
Yes	796 (89.06)	72 (91.41)			540 (65.50)	37 (58.29)		
Previous fracture, *n* (%)			*χ*^2^ = 42.824	< 0.001			*χ*^2^ = 5.346	0.021
No	927 (99.56)	79 (90.52)			948 (98.58)	60 (94.28)		
Yes	11 (0.44)	4 (9.48)			19 (1.42)	5 (5.72)		
BMI, kg/m^2^, mean (SE)	29.21 (0.25)	27.54 (0.84)	*t* = 1.88	0.080	29.74 (0.28)	27.29 (1.23)	*t* = 1.88	0.079
BMI, *n* (%)			*χ*^2^ = 3.165	0.075			*χ*^2^ = 3.591	0.058
Normal/underweight	220 (18.15)	24 (32.36)			243 (27.10)	20 (38.63)		
Overweight/obesity	718 (81.85)	59 (67.64)			724 (72.90)	45 (61.37)		
Waist circumference, cm, mean (SE)	106.09 (0.67)	103.12 (2.21)	*t* = 1.24	0.233	99.96 (0.62)	94.38 (2.36)	*t* = 2.25	0.040
Femoral neck BMD, g/cm^2^, mean (SE)	0.81 (0.01)	0.74 (0.01)	*t* = 4.35	< 0.001	0.73 (0.00)	0.64 (0.01)	*t* = 6.67	< 0.001
Mediterranean score, mean (SE)	4.60 (0.10)	4.20 (0.47)	*t* = 0.86	0.404	4.55 (0.13)	4.17 (0.29)	*t* = 1.45	0.168
*Mediterranean score, *n* (%)			*χ*^2^ = 0.094	0.759			*χ*^2^ = 2.300	0.129
< Median	307 (36.62)	30 (39.72)			317 (33.63)	25 (43.85)		
≥ Median	631 (63.38)	53 (60.28)			650 (66.37)	40 (56.15)		
Physical activity, MET·min, mean (SE)	871.69 (63.99)	580.92 (100.42)	*t* = 2.52	0.023	453.85 (40.22)	344.51 (71.34)	*t* = 1.31	0.209
HEI-2010 score, mean (SE)	52.32 (0.87)	50.41 (2.23)	*t* = 0.75	0.463	54.77 (0.59)	55.12 (2.03)	*t* = − 0.20	0.843
Diabetes, *n* (%)			*χ*^2^ = 0.557	0.455			*χ*^2^ = 0.843	0.359
No	350 (49.82)	32 (41.54)			376 (46.74)	23 (37.82)		
Yes	588 (50.18)	51 (58.46)			591 (53.26)	42 (62.18)		
Parental fracture, *n* (%)			*χ*^2^ = 0.023	0.879			*χ*^2^ = 0.139	0.710
No	862 (92.47)	73 (91.86)			869 (87.09)	58 (89.86)		
Yes	76 (7.53)	10 (8.14)			98 (12.91)	7 (10.14)		
Glucocorticoid use, *n* (%)			*χ*^2^ = 0.000	0.998			*χ*^2^ = 4.151	0.042
No	917 (97.14)	79 (97.15)			943 (97.33)	63 (99.65)		
Yes	21 (2.86)	4 (2.85)			24 (2.67)	2 (0.35)		
Calcium, mg, mean (SE)	1111.61 (23.70)	1235.55 (95.94)	*t* = − 1.28	0.220	1154.87 (39.04)	1374.09 (86.98)	*t* = − 2.18	0.046
Total energy intake, kcal, mean (SE)	2248.43 (35.12)	2268.87 (154.45)	*t* = − 0.14	0.888	1746.50 (27.29)	1890.88 (103.35)	*t* = − 1.44	0.171
Menopause, *n* (%)								
No					88 (10.18)	0 (0.00)		
Yes					879 (89.82)	65 (100.00)		
Non-carotenoid vitamin A, µg, mean (SE)	458.14 (13.73)	490.69 (76.21)	*t* = − 0.44	0.668	405.72 (24.40)	496.17 (41.20)	*t* = − 1.77	0.098
α-Carotene, µg, mean (SE)	426.29 (18.14)	459.10 (83.56)	*t* = − 0.38	0.709	535.48 (38.94)	497.81 (82.52)	*t* = 0.45	0.662
β-Carotene, µg, mean (SE)	2419.46 (130.71)	2250.69 (183.19)	*t* = 0.62	0.542	2742.27 (154.71)	2206.25 (203.57)	*t* = 1.98	0.066
β-Cryptoxanthin, µg, mean (SE)	100.17 (9.01)	147.60 (69.15)	*t* = − 0.69	0.501	99.49 (10.99)	79.75 (17.23)	*t* = 0.94	0.361
Lycopene, µg, mean (SE)	5577.98 (623.88)	7308.50 (1668.02)	*t* = − 0.99	0.338	4424.13 (245.52)	3402.50 (785.99)	*t* = 1.17	0.261
Lutein and zeaxanthin, µg, mean (SE)	1854.80 (92.87)	1562.27 (187.67)	*t* = 1.64	0.123	1996.13 (141.11)	1730.81 (260.51)	*t* = 0.88	0.395
Carotenoid vitamin A, mean (SE)	223.56 (10.99)	212.84 (17.32)	*t* = 0.46	0.651	254.98 (13.81)	207.92 (19.45)	*t* = 1.89	0.079
Total carotenoid, mean (SE)	10,378.70 (662.38)	11,728.16 (1827.07)	*t* = − 0.71	0.491	9797.50 (400.35)	7917.11 (833.09)	*t* = 1.78	0.095
Energy-adjusted α-carotene, mean (SE)	205.60 (11.47)	245.09 (54.97)	*t* = − 0.73	0.477	306.45 (14.47)	257.56 (49.85)	*t* = 1.00	0.333
Energy-adjusted β-carotene, mean (SE)	1.16 (0.08)	1.12 (0.10)	*t* = 0.31	0.759	1.60 (0.08)	1.23 (0.13)	*t* = 2.61	0.020
Energy-adjusted β-cryptoxanthin, mean (SE)	45.78 (3.96)	56.73 (21.37)	*t* = − 0.51	0.619	59.19 (4.85)	44.02 (8.32)	*t* = 1.53	0.147
Energy-adjusted lycopene, mean (SE)	2563.16 (273.40)	3076.09 (616.12)	*t* = − 0.77	0.455	2613.48 (169.55)	1856.27 (453.86)	*t* = 1.47	0.161
Energy-adjusted lutein and zeaxanthin, mean (SE)	897.77 (46.08)	774.29 (93.35)	*t* = 1.41	0.179	1172.77 (76.72)	1034.35 (198.64)	*t* = 0.65	0.525
Energy-adjusted carotenoid vitamin A, mean (SE)	107.17 (6.57)	106.30 (10.15)	*t* = 0.08	0.936	148.55 (6.79)	115.10 (12.37)	*t* = 2.57	0.021
Energy-adjusted total carotenoid, mean (SE)	4872.68 (318.71)	5276.92 (668.48)	*t* = − 0.56	0.582	5751.62 (244.86)	4422.56 (372.63)	*t* = 2.73	0.015

Notes: *SE*, standard error; *PIR*, poverty income ratio; *GED*, General Education Development; *BMI*, body mass index; *BMD*, bone mineral density; *MET*, physical activity; *HEI*, the Healthy Eating Index

^*^Median value in males was 3.77, and in females was 3.82

Carotenoid vitamin A = 1/12 × β-carotene + 1/24 × α-carotene + 1/24 × β-cryptoxanthin

Total carotenoid = α-carotene + β-carotene + β-cryptoxanthin + lycopene + lutein and zeaxanthin

Energy-adjusted carotenoids (µg/1000 kcal) = dietary carotenoid intake/energy intake × 1000

### Distribution of dietary carotenoids

Table 2 shows the average levels of five dietary carotenoids in men and women. The average levels of α-carotene, β-carotene, lutein and zeaxanthin, as well as carotenoid vitamin A were higher in women than those in men (532.11 vs. 425.98 µg, 2799.61 vs. 2385.89 µg, 1989.45 vs. 1815.78 µg, and 259.65 vs. 220.9). The average levels of β-cryptoxanthin, lycopene, and total carotenoid were higher in men than those in women (103.75 vs. 100.34 µg, 5690.55 vs. 4338.52 µg, and 10,422 vs. 9760.04). After adjusting for energy, only men had a higher lycopene level than women (2592.37 vs. 2561.69 µg/1000 kcal).

**Table 2:** Distribution of dietary carotenoids by quartile

Variables	Male	Female
α-Carotene, µg, mean (SE)	425.98 (35.12)	532.11 (59.05)
β-Carotene, µg, mean (SE)	2385.89 (162.35)	2799.61 (183.81)
β-Cryptoxanthin, µg, mean (SE)	103.75 (11.91)	100.34 (11.01)
Lycopene, µg, mean (SE)	5690.55 (556.65)	4338.52 (282.77)
Lutein and zeaxanthin, µg, mean (SE)	1815.78 (111.03)	1989.45 (119.62)
Carotenoid vitamin A, mean (SE)	220.9 (14.47)	259.65 (17.18)
Total carotenoid, mean (SE)	10,422 (609.38)	9760.04 (432.89)
Energy-adjusted α-carotene, mean (SE)	207.58 (17.94)	302.89 (29.7)
Energy-adjusted β-carotene, mean (SE)	1.15 (0.08)	1.63 (0.1)
Energy-adjusted β-cryptoxanthin, mean (SE)	46.52 (4.87)	59.69 (5.43)
Energy-adjusted lycopene, mean (SE)	2592.37 (239.72)	2561.69 (176.17)
Energy-adjusted lutein and zeaxanthin, mean (SE)	880.91 (59.52)	1173.96 (66.77)
Energy-adjusted carotenoid vitamin A, mean (SE)	106.33 (6.91)	150.94 (9.13)
Energy-adjusted total carotenoid, mean (SE)	4876.29 (267.83)	5728.23 (250.5)

Notes: *SE* standard error

Carotenoid vitamin A = 1/12 × β-carotene + 1/24 × α-carotene + 1/24 × β-cryptoxanthin

Total carotenoid = α-carotene + β-carotene + β-cryptoxanthin + lycopene + lutein and zeaxanthin

Energy-adjusted carotenoids (µg/1000 kcal) = dietary carotenoid intake/energy intake × 1000

### Correlations among dietary carotenoids

The results showed a highly positive correlation between energy-adjusted α-carotene and β-carotene in both the man group (*r* = 0.5800, *P* < 0.001) and feman group (*r* = 0.6563, *P* < 0.001), indicating that these two dietary carotenoids could not be mutually adjusted in a multivariable model, and only one of them could be adjusted in the variable adjustment for the other carotenoids (Table 3).

**Table 3:** Correlations among dietary carotenoids

Energy-adjusted carotenoids	Male					Female				
	α-Carotene	β-Carotene	β-Cryptoxanthin	Lycopene	Lutein and zeaxanthin	α-Carotene	β-Carotene	β-Cryptoxanthin	Lycopene	Lutein and zeaxanthin
α-Carotene	1.000					1.000				
β-Carotene	0.5800***	1.000				0.6563***	1.000			
β-Cryptoxanthin	0.0378	0.0904**	1.000			0.0544	0.0792**	1.000		
Lycopene	0.0048	0.0484	0.0800*	1.000		0.0115	0.0495	0.0425	1.000	
Lutein and zeaxanthin	0.1915***	0.3146***	0.0631*	0.0735*	1.000	0.1094	0.5407***	0.1058***	0.0111	1.000

Notes: Carotenoid vitamin A = 1/12 × β-carotene + 1/24 × α-carotene + 1/24 × β-cryptoxanthin

Total carotenoid = α-carotene + β-carotene + β-cryptoxanthin + lycopene + lutein and zeaxanthin

Energy-adjusted carotenoids (µg/1000 kcal) = dietary carotenoid intake/energy intake × 1000

^***^P < 0.001, **P < 0.01, *P < 0.05

### Association between dietary carotenoid intake and vertebral fracture

Univariable logistic regression showed that age, race, PIR, a history of fracture, and femoral neck BMD were significantly associated with vertebral fracture in men, and age, race, a history of fracture, and femoral neck BMD were significantly associated with vertebral fractures in women (Table 4). After adjusting for selected covariates, multivariable logistic regression demonstrated that elevated intake levels of energy-adjusted β-carotene (OR = 0.75, 95% CI 0.57–0.99, *P* = 0.046), energy-adjusted carotenoid vitamin A (OR = 0.75, 95% CI 0.57–0.99, *P* = 0.045), and energy-adjusted total carotenoid (OR = 0.72, 95% CI 0.56–0.94, *P* = 0.015) were significantly associated with a lower risk of vertebral fracture in women. After controlling for other carotenoids, only the negative association between energy-adjusted β-carotene and increased odds of vertebral fracture was significant in women (OR = 0.77, 95% CI 0.60–0.99, *P* = 0.047) (Table 5).

**Table 4:** Weighted univariable logistic regression to select adjustment variables

Variables	Male		Female	
	OR (95% CI)	*P*	OR (95% CI)	*P*
Age	1.97 (1.41–2.73)	< 0.001	1.85 (1.33–2.58)	< 0.001
Race				
Mexican American	Ref		Ref	
Non-Hispanic Black	0.24 (0.06–0.99)	0.048	1.57 (0.48–5.16)	0.457
Non-Hispanic White	0.58 (0.18–1.91)	0.375	2.91 (1.03–8.17)	0.043
Other Hispanic	0.23 (0.02–2.45)	0.222	1.52 (0.27–8.49)	0.637
Other race	1.50 (0.34–6.57)	0.591	2.21 (0.37–13.22)	0.385
PIR	0.67 (0.50–0.88)	0.005	0.85 (0.58–1.23)	0.385
Education				
College graduate	Ref		Ref	
High school/GED	1.22 (0.48–3.09)	0.673	0.75 (0.39–1.45)	0.395
Less than high school	0.84 (0.27–2.55)	0.756	1.08 (0.34–3.46)	0.898
Some college	1.28 (0.48–3.44)	0.622	0.46 (0.20–1.06)	0.069
Smoking				
No	Ref		Ref	
Yes	2.10 (0.90–4.91)	0.088	0.88 (0.34–2.28)	0.785
Alcohol consumption				
No	Ref		Ref	
Yes	1.32 (0.51–3.42)	0.564	0.74 (0.36–1.53)	0.420
Previous fracture				
No	Ref		Ref	
Yes	23.51 (5.26–105.00)	< 0.001	4.17 (1.22–14.23)	0.023
BMI				
Normal/underweight	Ref		Ref	
Overweight/obesity	0.43 (0.18–1.03)	0.058	0.66 (0.39–1.11)	0.117
Waist circumference	0.77 (0.52–1.14)	0.196	0.71 (0.50–1.01)	0.056
Femoral neck BMD	0.51 (0.37–0.71)	< 0.001	0.38 (0.27–0.54)	< 0.001
Mediterranean score				
< Median	Ref		Ref	
≥ Median	0.86 (0.37–1.98)	0.723	0.66 (0.38–1.17)	0.154
Physical activity	0.64 (0.41–1.01)	0.055	0.53 (0.15–1.90)	0.330
HEI-2010	0.93 (0.66–1.32)	0.692	1.00 (0.79–1.26)	0.993
Diabetes				
No	Ref		Ref	
Yes	1.38 (0.58–3.31)	0.467	1.46 (0.70–3.03)	0.311
Parental fracture				
No	Ref		Ref	
Yes	1.08 (0.36–3.21)	0.896	0.75 (0.18–3.15)	0.696
Glucocorticoid use				
No	Ref		Ref	
Yes	1.22 (0.24–6.18)	0.814	0.50 (0.08–3.18)	0.462
Calcium	1.24 (0.94–1.64)	0.124	1.26 (1.00–1.58)	0.054
Non-carotenoid vitamin A	1.11 (0.75–1.64)	0.612	1.19 (0.99–1.44)	0.069
Total energy intake	1.02 (0.68–1.54)	0.920	1.24 (0.94–1.65)	0.134

Notes: *OR*, odds ratio; *CI*, confidence interval; *Ref*, reference; *PIR*, poverty income ratio; *GED*, General Education Development; *BMI*, body mass index; *BMD*, bone mineral density; *HEI*, the Healthy Eating Index

**Table 5:** Association between dietary carotenoid intake and vertebral fracture

Variables	Univariable model		Multivariable model^1^		Multivariable model^2^	
	OR (95% CI)	*P*	OR (95% CI)	*P*	OR (95% CI)	*P*
**Male**						
α-Carotene	1.05 (0.85–1.29)	0.654	1.03 (0.82–1.30)	0.787	1.10 (0.87–1.40)	0.424
β-Carotene	0.94 (0.79–1.13)	0.524	0.97 (0.84–1.11)	0.617	1.04 (0.90–1.20)	0.562
β-Cryptoxanthin	1.12 (0.99–1.28)	0.078	1.11 (0.98–1.26)	0.115	1.07 (0.93–1.23)	0.347
Lycopene	1.18 (0.87–1.60)	0.275	1.25 (0.94–1.67)	0.122	1.22 (0.94–1.59)	0.140
Lutein and zeaxanthin	0.85 (0.68–1.07)	0.161	0.84 (0.65–1.08)	0.177	0.78 (0.58–1.05)	0.104
Carotenoid vitamin A	0.96 (0.81–1.14)	0.646	0.98 (0.86–1.13)	0.793	1.06 (0.92–1.23)	0.412
Total carotenoid	1.13 (0.82–1.56)	0.463	1.17 (0.89–1.54)	0.253		
Energy-adjusted α-carotene	1.10 (0.89–1.37)	0.373	1.05 (0.80–1.37)	0.737	1.15 (0.85–1.54)	0.362
Energy-adjusted β-carotene	0.97 (0.80–1.18)	0.774	0.95 (0.75–1.21)	0.685	1.05 (0.80–1.38)	0.701
Energy-adjusted β-cryptoxanthin	1.20 (0.81–1.78)	0.362	1.15 (0.78–1.69)	0.484	1.11 (0.73–1.69)	0.615
Energy-adjusted lycopene	1.14 (0.83–1.57)	0.419	1.17 (0.90–1.52)	0.252	1.17 (0.90–1.52)	0.252
Energy-adjusted lutein and zeaxanthin	0.89 (0.74–1.07)	0.217	0.84 (0.65–1.10)	0.206	0.78 (0.57–1.06)	0.113
Energy-adjusted carotenoid vitamin A	0.99 (0.83–1.20)	0.958	0.97 (0.76–1.24)	0.807	1.08 (0.82–1.43)	0.568
Energy-adjusted total carotenoid	1.09 (0.81–1.48)	0.565	1.08 (0.83–1.40)	0.561		
**Female**						
α-Carotene	0.96 (0.82–1.12)	0.593	0.97 (0.81–1.18)	0.791	0.94 (0.77–1.15)	0.560
β-Carotene	0.78 (0.61–0.98)	0.036	0.82 (0.64–1.06)	0.127	0.79 (0.62–1.01)	0.057
β-Cryptoxanthin	0.74 (0.24–2.29)	0.606	0.58 (0.16–2.09)	0.407	0.63 (0.17–2.39)	0.495
Lycopene	0.84 (0.57–1.23)	0.362	0.86 (0.57–1.28)	0.456	0.81 (0.52–1.25)	0.331
Lutein and zeaxanthin	0.83 (0.52–1.31)	0.411	0.89 (0.57–1.40)	0.617	0.83 (0.50–1.37)	0.466
Carotenoid vitamin A	0.79 (0.63–1.00)	0.051	0.83 (0.65–1.07)	0.150	0.79 (0.60–1.04)	0.090
Total carotenoid	0.75 (0.54–1.05)	0.093	0.79 (0.56–1.11)	0.168		
Energy-adjusted α-carotene	0.87 (0.64–1.17)	0.360	0.88 (0.63–1.24)	0.478	0.92 (0.67–1.27)	0.603
Energy-adjusted β-carotene	0.71 (0.56–0.92)	0.008	0.75 (0.57–0.99)	0.046	0.77 (0.60–0.99)	0.047
Energy-adjusted β-cryptoxanthin	0.60 (0.19–1.91)	0.388	0.39 (0.11–1.37)	0.141	0.46 (0.12–1.71)	0.245
Energy-adjusted lycopene	0.79 (0.50–1.23)	0.291	0.80 (0.53–1.22)	0.308	0.83 (0.52–1.31)	0.421
Energy-adjusted lutein and zeaxanthin	0.87 (0.57–1.34)	0.531	0.90 (0.57–1.44)	0.665	0.98 (0.63–1.51)	0.914
Energy-adjusted carotenoid vitamin A	0.72 (0.56–0.93)	0.010	0.75 (0.57–0.99)	0.045	0.75 (0.56–1.02)	0.070
Energy-adjusted total carotenoid	0.71 (0.54–0.92)	0.010	0.72 (0.56–0.94)	0.015		

Notes: Carotenoid vitamin A = 1/12 × β-carotene + 1/24 × α-carotene + 1/24 × β-cryptoxanthin

Total carotenoid = α-carotene + β-carotene + β-cryptoxanthin + lycopene + lutein and zeaxanthin

Energy-adjusted carotenoids (µg/1000 kcal) = dietary carotenoid intake/energy intake × 1000

^1^Carotenoids not adjusted for each other, multivariable models adjusted for age, race, PIR, previous fracture, femoral neck BMD in the male group, and that adjusted for age, race, previous fracture, and femoral neck BMD in the female group

^2^Carotenoids adjusted for each other on the basis of Multivariable model^1^, and considering the correlation between α-carotene and β-carotene, only adjusted for α-carotene

In addition, sensitivity analyses were performed to reclassify the non-vertebral and vertebral fracture groups to account for the effect of mild deformity (Supplementary Table 3). The results showed that after adjusting for the selected covariates, elevated intake levels of β-carotene (OR = 0.76, 95% CI 0.59–0.98, *P* = 0.036), carotenoid vitamin A (OR = 0.76, 95% CI 0.58–0.99, *P* = 0.046), energy-adjusted β-carotene (OR = 0.70, 95% CI 0.51–0.95, *P* = 0.021), and energy-adjusted carotenoid vitamin A (OR = 0.68, 95% CI 0.49–0.93, *P* = 0.016) were all significantly associated with a reduced risk of vertebral fracture in women. After controlling for other carotenoids, elevated dietary intakes of carotenoid vitamin A (OR = 0.69, 95% CI 0.50–0.97, *P* = 0.032), energy-adjusted β-carotene (OR = 0.11, 95% CI 0.01–0.83, *P* = 0.032), and energy-adjusted carotenoid vitamin A (OR = 0.62, 95% CI 0.41–0.93, *P* = 0.020) were significantly associated with a reduced risk of vertebral fracture in women.

### Association between dietary carotenoid intake and vertebral fracture in subgroups

**Fig. 2:**
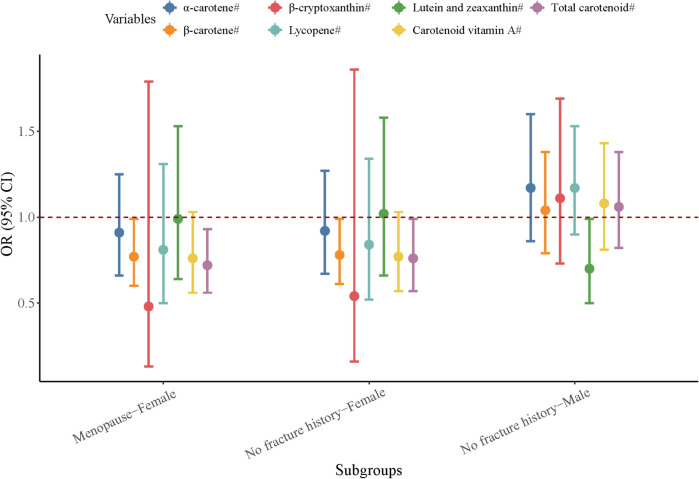
Association between energy-adjusted β-carotene intake and energy-adjusted total carotenoid

The sample size of the population with a history of fracture and premenopausal women was both too small to allow subgroup analysis. For men without a history of fractures, higher energy-adjusted intake of lutein and zeaxanthin was associated with a significantly lower risk of vertebral fracture (OR = 0.70, 95% CI 0.50–0.99, *P* = 0.048). For women without a history of fracture, elevated intake levels of energy-adjusted β-carotene (OR = 0.78, 95% CI 0.61–0.99, *P* = 0.047) and energy-adjusted total carotenoid (OR = 0.76, 95% CI 0.57–0.99, *P* = 0.046) were associated with a decreased risk of vertebral fracture. Additionally, among post-menopause women, there was a negative association between energy-adjusted β-carotene intake (OR = 0.77, 95% CI 0.60–0.99, *P* = 0.048) and energy-adjusted total carotenoid (OR = 0.72, 95% CI 0.56–0.93, *P* = 0.010) with an increased risk of vertebral fracture (Fig. 2).

## Discussion

The burden of vertebral fracture is substantial, with an estimated prevalence of 25–50% in people aged over 50 years, which in some research is higher than hip or wrist fractures [17]. To our knowledge, this study is the first to investigate the association between dietary carotenoid intake and vertebral fracture in individuals aged ≥ 50 years based on data from the NHANES database. We found that higher intakes of β-carotene, carotenoid vitamin A, and total carotenoid were associated with a significantly decreased risk of vertebral fracture in feman, indicating that increasing carotenoid intake, especially β-carotene, may help lower the potential risk of vertebral fracture in women.

Knapik et al. [18] reported that the risk of hip fracture was lowered by increasing the intake of β-carotene, or α-carotene. In another study, greater lycopene intake was associated with reduced risk of hip fracture and non-vertebral fracture, but not with α-carotene, β-carotene, β-cryptoxanthin, or lutein and zeaxanthin [19]. A previous meta-analysis found that β-cryptoxanthin intake was inversely associated with the risk of osteoporosis and hip fractures [20]. In Gao et al.’s study, they suggested that β-carotene may improve BMD and reduce the risk of osteoporosis/fracture, but these effects may vary by gender and race [21]. For the relationship between individual carotenoids and vertebral fracture, the current study demonstrated that dietary intakes of β-carotene, carotenoid vitamin A, and total carotenoid were inversely associated with the risk of vertebral fracture in women, while different kinds of carotenoids were not in significantly associated with the risk of vertebral fracture in men. Several biological mechanisms have been proposed to help explain the association between β-carotene and vertebral fracture. Adequate intake of vitamin A (including β-carotene) is essential for normal physiological activities of the human body and can affect the growth hormone axis [22, 23]. The antioxidant β-carotene contributes to defense against reactive oxygen species in the body, and oxidative stress is considered to play a significant role in the development of several chronic diseases, including fractures [24]. β-Carotene enhances osteoclastogenesis and reduces osteoblast apoptosis by stabilizing the β-catenin signaling pathway, leading to a decrease in bone resorption [25, 26]. Also, carotenoids may interfere with growth factor receptor signaling via regulating IGF-1/IGFBP3, which is related to cognitive function [27]. Impaired cognitive function is a known risk factor for falls and fractures [28].

Different carotenoids may have different associations with vertebral fracture risk [29–31]. One explanation for the non-significant association of α-carotene, β-cryptoxanthin, lycopene, and lutein and zeaxanthin with vertebral fractures may be that the range and magnitude of intake of different carotenoids are different. In other words, α-carotene, β-carotene, β-cryptoxanthin, lycopene, and lutein and zeaxanthin are greatly sourced from different fruits and vegetables [29], and insufficient intakes of individuals may make the associations between specific carotenoids and the risk of vertebral fracture insignificant. Some people may not develop vertebral fracture at the time of study, which might lead to the above insignificant associations. Additionally, differential absorption of different carotenoids may affect interpretation [32]. Herein, we explored the association of each carotenoid with vertebral fracture after adjusting for other carotenoids. The results showed a significant negative association between β-carotene intake and increased risk of vertebral fracture in women. This provided some information for further investigation on the prevention and treatment of vertebral fractures at the dietary level among this population.

Furthermore, we found that for men without a history of fracture, lutein and zeaxanthin intake was negatively associated with an increased risk of vertebral fracture, whereas higher β-carotene intake was associated with a decreased risk of vertebral fracture in women without a history of fracture. Dai et al. [33] revealed that high dietary intakes of α-carotene, β-carotene, and lutein and zeaxanthin were associated with a reduced risk of hip fracture in men, while no association was observed between dietary carotenoids and the hip fracture risk in women. Another previous study showed that plasma α-carotene and β-carotene were significantly associated with the risk of hip fracture in men [29]. Higher intake of β-cryptoxanthin was correlated with a lower risk of osteoporosis and remained significant in both men and women [20]. The difference between men and women in terms of the association between specific carotenoid intake and vertebral fracture may be attributed to the gender differences in carotenoid consumption or reporting [34]. Previous fracture history could increase the risk of vertebral fracture [35, 36], but the number of people with a history of fracture in our research was too small for subgroup analysis. It was also exhibited herein that among postmenopausal women, increased intakes of β-carotene and total carotenoid were associated with a significantly decreased odds of vertebral fracture. In fact, vertebral fractures increase with age in postmenopausal women. Published literature has suggested an overall prevalence between 10–20% and 35–40% in women ≥ 80 [37]. Maggio et al. [38] concluded that β-carotene levels were independently and inversely associated with estradiol in older women. Estradiol levels decline during menopause and progressively increase from the age of 65. In addition to estrogen-dependent cancers, β-carotene intake levels may be associated with fractures, with post-menopause being a potential risk factor [39]. Nevertheless, future studies with larger sample sizes are needed to clarify the association between carotenoid intake and vertebral fracture in individuals with a history of fracture or in premenopausal women.

This study utilized the nationally representative NHANES database to probe into the association between individual dietary carotenoids and vertebral fracture, which may provide certain reference for dietary prevention and treatment of vertebral fracture in middle-aged and elderly people. The association between the intake of β-carotene, carotenoid vitamin A, and total carotenoid with vertebral fracture should be popularized in women ≥ 50 years to promote the understanding of the role of dietary carotenoids in the management of potential vertebral fracture risk. Further studies are needed to verify whether dietary supplementation with β-carotene can reduce the risk of vertebral fracture. Several limitations should be mentioned in the result interpretation. First, due to the cross-sectional design, we could not determine the causal relationship between dietary carotenoids and vertebral fractures. Second, dietary carotenoid intake was obtained through a 24-h dietary recall interview, which may reflect only short-term dietary status and might be subject to recall bias. Besides, the range of osteoporotic fractures is much larger, but only hip fracture was included as “previous fracture,” which may affect the accuracy of the results. Third, if the disease studied in the cross-sectional study actually has an extended risk period, the prevalence odds ratio is generally a better approximation of the incidence density ratio, but it would have considerably overestimated the strength of the association, which is not always a good estimate of the true odds ratio. Finally, the NHANES provided data on a US population, which may limit the generalizability of our findings to other populations.

## Conclusion

The intake of total carotenoids, especially β-carotene, was negatively associated with the risk of vertebral fracture in women aged 50 years and older. This significant association was also found in women without a history of fracture. Future controlled trials should investigate the mechanism.

## Supplementary Information

Below is the link to the electronic supplementary material.

Supplementary file1 (DOCX 18 KB)

Supplementary file2 (DOCX 19 KB)

Supplementary file3 (DOCX 23 KB)

## Data Availability

The datasets used during the current study are available from the corresponding author on reasonable request.
