# Supplementary material for: Association between dietary carotenoid intake and vertebral fracture in people aged 50 years and older: a study based on the National Health and Nutrition Examination Survey
Source: Arch Osteoporos. 2025 Mar 15;20(1):39. doi: 10.1007/s11657-025-01508-5 (PMC11910422; doi:10.1007/s11657-025-01508-5)
Supplement: Supplementary file 1 — Supplementary file1 (DOCX 18 KB) [file 11657_2025_1508_MOESM1_ESM.docx]

**Supplementary Table 1 Variables with missing values and missing proportion**

| Variables | Numbers | Proportion (%) |
| --- | --- | --- |
| Smoking | 1 | 0.05 |
| Education | 1 | 0.05 |
| BMI | 11 | 0.54 |
| Waist circumference | 24 | 1.17 |
| Alcohol consumption | 51 | 2.48 |
| Femoral neck BMD | 137 | 6.67 |
| PIR | 149 | 7.26 |

Notes: BMI, Body Mass Index; BMD, bone mineral density; PIR, poverty income ratio.
