# Supplementary material for: Association between dietary carotenoid intake and vertebral fracture in people aged 50 years and older: a study based on the National Health and Nutrition Examination Survey
Source: Arch Osteoporos. 2025 Mar 15;20(1):39. doi: 10.1007/s11657-025-01508-5 (PMC11910422; doi:10.1007/s11657-025-01508-5)
Supplement: Supplementary file 2 — Supplementary file2 (DOCX 19 KB) [file 11657_2025_1508_MOESM2_ESM.docx]

**Supplementary Table 2 Sensitivity analysis for the data before and after imputation**

| Variables | After imputation (n=2053) | Before imputation (n=2053) | Statistics | *P* |
| --- | --- | --- | --- | --- |
| PIR, Mean (SE) | 3.25 (0.12) | 3.27 (0.13) | t=-0.99 | 0.340 |
| Education, n (%) |  |  | χ^2^=2.999 | 0.392 |
| College graduate | 511 (33.89) | 511 (33.89) |  |  |
| High school/GED | 473 (20.06) | 473 (20.06) |  |  |
| Less than high school | 471 (14.12) | 470 (14.12) |  |  |
| Some college | 598 (31.93) | 598 (31.94) |  |  |
| Smoking, n (%) |  |  | χ^2^=0.965 | 0.326 |
| No | 1042 (52.12) | 1041 (52.11) |  |  |
| Yes | 1011 (47.88) | 1011 (47.89) |  |  |
| Alcohol consumption, n (%) |  |  | χ^2^=0.540 | 0.462 |
| No | 608 (23.24) | 591 (23.31) |  |  |
| Yes | 1445 (76.76) | 1411 (76.69) |  |  |
| BMI, kg/m^2^, Mean (SE) | 29.34 (0.16) | 29.32 (0.16) | t=1.53 | 0.147 |
| Waist circumference, cm, Mean (SE) | 102.63 (0.44) | 102.61 (0.43) | t=0.89 | 0.388 |
| Femoral neck BMD, gm/cm^2^, Mean (SE) | 0.76 (0.00) | 0.76 (0.01) | t=1.43 | 0.174 |

Notes: PIR, poverty income ratio; SE, standard error; GED, General Education Development; BMI, Body Mass Index; BMD, bone mineral density.
