# Supplementary material for: Association between dietary carotenoid intake and vertebral fracture in people aged 50 years and older: a study based on the National Health and Nutrition Examination Survey
Source: Arch Osteoporos. 2025 Mar 15;20(1):39. doi: 10.1007/s11657-025-01508-5 (PMC11910422; doi:10.1007/s11657-025-01508-5)
Supplement: Supplementary file 3 — Supplementary file3 (DOCX 23 KB) [file 11657_2025_1508_MOESM3_ESM.docx]

**Supplementary Table 3 Association between dietary carotenoid intake and vertebral fracture (sensitivity analysis)**

| Variables | Univariable model | | Multivariable model^1^ | | Multivariable model^2^ | |
| --- | --- | --- | --- | --- | --- | --- |
|  | OR (95% CI) | *P* | OR (95% CI) | *P* | OR (95% CI) | *P* |
| **Male** |  |  |  |  |  |  |
| α-carotene | 0.87 (0.59-1.27) | 0.469 | 0.87 (0.59-1.27) | 0.461 | 0.92 (0.59-1.44) | 0.718 |
| β-carotene | 0.95 (0.68-1.33) | 0.768 | 0.98 (0.77-1.25) | 0.859 | 1.11 (0.81-1.52) | 0.507 |
| β-cryptoxanthin | 0.77 (0.38-1.59) | 0.485 | 0.71 (0.30-1.69) | 0.435 | 0.62 (0.22-1.72) | 0.354 |
| Lycopene | 1.10 (0.81-1.51) | 0.535 | 1.18 (0.90-1.55) | 0.228 | 1.15 (0.88-1.52) | 0.308 |
| Lutein and zeaxanthin | 0.84 (0.60-1.18) | 0.317 | 0.82 (0.56-1.21) | 0.315 | 0.81 (0.53-1.25) | 0.345 |
| Carotenoid vitamin A | 0.94 (0.67-1.32) | 0.710 | 0.96 (0.74-1.24) | 0.754 | 1.04 (0.75-1.44) | 0.801 |
| Total carotenoid | 1.04 (0.71-1.52) | 0.849 | 1.10 (0.82-1.47) | 0.534 |  |  |
| Energy-adjusted α-carotene | 0.93 (0.64-1.37) | 0.719 | 0.88 (0.59-1.31) | 0.526 | 0.95 (0.57-1.58) | 0.843 |
| Energy-adjusted β-carotene | 0.96 (0.65-1.41) | 0.824 | 0.92 (0.66-1.28) | 0.618 | 1.11 (0.72-1.70) | 0.638 |
| Energy-adjusted β-cryptoxanthin | 0.64 (0.23-1.74) | 0.379 | 0.49 (0.12-1.95) | 0.311 | 0.48 (0.10-2.18) | 0.340 |
| Energy-adjusted lycopene | 1.04 (0.74-1.46) | 0.833 | 1.08 (0.84-1.37) | 0.548 | 1.13 (0.84-1.51) | 0.424 |
| Energy-adjusted lutein and zeaxanthin | 0.88 (0.65-1.19) | 0.412 | 0.82 (0.54-1.24) | 0.344 | 0.84 (0.54-1.30) | 0.436 |
| Energy-adjusted carotenoid vitamin A | 0.95 (0.64-1.40) | 0.788 | 0.91 (0.64-1.28) | 0.570 | 1.01 (0.65-1.58) | 0.963 |
| Energy-adjusted total carotenoid | 0.98 (0.67-1.44) | 0.916 | 0.97 (0.74-1.29) | 0.856 |  |  |
| **Female** |  |  |  |  |  |  |
| α-carotene | 0.86 (0.64-1.15) | 0.305 | 0.88 (0.67-1.16) | 0.373 | 0.89 (0.67-1.17) | 0.395 |
| β-carotene | 0.66 (0.42-1.02) | 0.063 | 0.76 (0.59-0.98) | 0.036 | 0.74 (0.54-1.02) | 0.064 |
| β-cryptoxanthin | 0.53 (0.05-5.34) | 0.588 | 0.26 (0.02-3.16) | 0.290 | 0.23 (0.02-3.38) | 0.283 |
| Lycopene | 1.06 (0.76-1.49) | 0.715 | 1.16 (0.82-1.65) | 0.402 | 1.21 (0.80-1.85) | 0.367 |
| Lutein and zeaxanthin | 0.81 (0.51-1.29) | 0.375 | 0.91 (0.59-1.40) | 0.664 | 0.98 (0.70-1.38) | 0.921 |
| Carotenoid vitamin A | 0.67 (0.43-1.04) | 0.074 | 0.76 (0.58-0.99) | 0.046 | 0.69 (0.50-0.97) | 0.032 |
| Total carotenoid | 0.88 (0.57-1.35) | 0.555 | 1.00 (0.69-1.45) | 0.984 |  |  |
| Energy-adjusted α-carotene | 0.75 (0.52-1.07) | 0.112 | 0.75 (0.52-1.10) | 0.142 | 0.79 (0.53-1.18) | 0.254 |
| Energy-adjusted β-carotene | 0.62 (0.39-0.99) | 0.046 | 0.70 (0.51-0.95) | 0.021 | 0.11 (0.01-0.83) | 0.032 |
| Energy-adjusted β-cryptoxanthin | 0.41 (0.06-2.74) | 0.354 | 0.14 (0.02-1.22) | 0.075 | 0.12 (0.01-1.17) | 0.068 |
| Energy-adjusted lycopene | 1.09 (0.76-1.58) | 0.629 | 1.13 (0.80-1.59) | 0.493 | 1.20 (0.82-1.77) | 0.344 |
| Energy-adjusted lutein and zeaxanthin | 0.89 (0.60-1.31) | 0.548 | 0.91 (0.59-1.42) | 0.682 | 1.06 (0.73-1.55) | 0.760 |
| Energy-adjusted carotenoid vitamin A | 0.62 (0.39-0.98) | 0.043 | 0.68 (0.49-0.93) | 0.016 | 0.62 (0.41-0.93) | 0.020 |
| Energy-adjusted total carotenoid | 0.91 (0.56-1.46) | 0.690 | 0.98 (0.62-1.55) | 0.936 |  |  |

Notes: vertebral fracture was defined as grade 2 and 3.

Carotenoid vitamin A = 1/12 * β-carotene + 1/24 * α-carotene + 1/24 * β-cryptoxanthin;

Total carotenoid = α-carotene + β-carotene + β-cryptoxanthin + Lycopene + Lutein and zeaxanthin.

Energy-adjusted carotenoids (μg/1000 kcal) = dietary carotenoid intake / energy intake × 1000.

^1^ Carotenoids not adjusted for each other, multivariable models adjusted for age, race, PIR, previous fracture, femoral neck BMD in male group, and that adjusted for age, race, previous fracture, and femoral neck BMD in female group;

^2^ Carotenoids adjusted for each other on the basis of Multivariable model^1^, and considering the correlation between α-carotene and β-carotene, only adjusted for α-carotene.
